# Proton and Oxygen-Ion Conductivities of Hexagonal Perovskite Ba_5_In_2_Al_2_ZrO_13_

**DOI:** 10.3390/ma15113944

**Published:** 2022-06-01

**Authors:** Roman Andreev, Daniil Korona, Irina Anokhina, Irina Animitsa

**Affiliations:** 1 Institute of Natural Sciences and Mathematics, Ural Federal University, 620002 Yekaterinburg, Russia; andreev.roman@urfu.ru (R.A.); d.v.korona@urfu.ru (D.K.); ianokhina24@gmail.com (I.A.); 2 Institute of High Temperature Electrochemistry of the Ural Branch of the Russian Academy of Sciences, 620990 Yekaterinburg, Russia

**Keywords:** perovskite hexagonal structure, proton conductors, transport properties, hydration

## Abstract

The hexagonal perovskite Ba_5_In_2_Al_2_ZrO_13_ and In^3+^-doped phase Ba_5_In_2.1_Al_2_Zr_0.9_O_12.95_ were prepared by the solid-state synthesis method. The introduction of indium in the Zr-sublattice was accompanied by an increase in the unit cell parameters: *a* = 5.967 Å, *c* = 24.006 Å vs. *a* = 5.970 Å, *c* = 24.011 Å for doped phase (space group of *P6_3_/mmc*). Both phases were capable of incorporating water from the gas phase. The ability of water incorporation was due to the presence of oxygen deficient blocks in the structure, and due to the introduction of oxygen vacancies during doping. According to thermogravimetric (TG) measurements the compositions of the hydrated samples corresponded to Ba_5_In_2_Al_2_ZrO_12.7_(OH)_0.6_ and Ba_5_In_2.1_Al_2_Zr_0.9_O_12.54_(OH)_0.82_. The presence of different types of OH^−^-groups in the structure, which participate in different hydrogen bonds, was confirmed by infrared (IR) investigations. The measurements of bulk conductivity by the impedance spectroscopy method showed that In^3+^-doping led to an increase in conductivity by 0.5 order of magnitude in wet air (*p*H_2_O = 1.92·10^−2^ atm); in this case, the activation energies decreased from 0.27 to 0.19 eV. The conductivity−pO_2_ measurements showed that both the phases were dominant proton conductors at T < 500 °C in wet conditions. The composition Ba_5_In_2.1_Al_2_Zr_0.9_O_12.95_ exhibited a proton conductivity ~10^−4^ S·cm^−1^ at 500 °C. The analysis of partial (O^2−^, H^+^, h^•^) conductivities of the investigated phases has been carried out. Both phases in dry air (*p*H_2_O = 3.5·10^−5^ atm) showed a mixed (oxygen-ion and hole) type of conductivity. The obtained results indicated that the investigated phases of Ba_5_In_2_Al_2_ZrO_13_ and Ba_5_In_2.1_Al_2_Zr_0.9_O_12.95_ might be promising proton-conducting oxides in the future applications in electrochemical devices, such as solid oxide fuel cells. Further modification of the composition and search for the optimal dopant concentrations can improve the H^+^-conductivity.

## 1. Introduction

Oxygen-ionic and proton electrolytes based on complex oxides have been extensively studied because of its potential for practical applications [1,2,3,4,5,6], and among various types of applications, the use of these materials in electrochemical devices, especially in solid oxide fuel cells (SOFCs), is the most important and most requested due to the development of clean, sustainable, and highly efficient energy conversion and storage technologies [7,8].

Proton-conducting oxides as electrolyte materials play a key role in the development of low- and intermediate- temperature SOFCs [3,9,10]. However, in addition to high proton conductivity, such electrolytes must have a set of other necessary properties, for example, chemical inertness to electrode materials, compatibility in terms of CTE, chemical resistance to CO_2_, etc. [11,12,13]. Therefore, materials science research has been focused on the discovery and development of novel and more efficient electrolyte material based on complex oxides with proton conductivity deserves significant research attention. As usual, attention has been focused on the perovskite materials, and the largest number of studies is devoted to doped phases based on barium cerates and zirconates—BaCeO_3_ and BaZrO_3_ [14].

In such systems, acceptor doping makes it possible to create oxygen vacancies, which, in a wet atmosphere, are filled with oxygen from a water molecule (as a result of dissociative adsorption), and OH^−^-groups are formed [15,16,17]. Thus, proton defects are formed, which in the framework of the quasi-chemical formalism are considered as an impurity [18]:

(1) $$H_{2}O+V_{O}^{••}+O_{O}^{\times }↔2{\left(\mathrm{OH}\right)}_{O}^{•},$$

where V_O_^••^ is oxygen vacancy, O_O_^×^ is oxygen at a regular oxygen site, and (OH)_O_^•^ is a hydroxyl group on the oxygen site, respectively.

However, there is another class of oxide materials in which oxygen vacancies are genetically present (without acceptor doping), such as brownmillerites [19,20,21,22,23,24,25,26] or oxygen-deficient double perovskites [27,28,29,30,31,32,33,34,35]. In such systems, polyhedra with a coordination number less than 6 (such as tetrahedra or pyramids) are present. Therefore, for such structures, it is possible to restore the coordination polyhedron to an octahedron due to dissociative adsorption of water vapor and the participation of OH^−^-groups in the coordination of the cation. The oxygen deficiency in such structures is significant; accordingly, the concentration of water (OH^−^-groups) in the structure can also reach the same high values. In this aspect, water becomes part of the chemical formula of the substance (for example, Ba_2_In_2_O_5_·H_2_O≡Ba_2_In_2_O_4_(OH)_2_), but due to the convenience of the quasi-chemical approach, it is also used for such systems, and the hydration reaction is given as [22,36]:

(2) $$H_{2}O+2O_{O}^{\times }+V_{O}^{\times }↔2{\left(\mathrm{OH}\right)}_{O}^{•}+O_{V_{O}}^{\prime \prime }$$

or

(3) $$H_{2}O+O_{O}^{\times }+V_{O}^{\times }↔{\left(\mathrm{OH}\right)}_{O}^{•}+{\left(\mathrm{OH}\right)}_{V_{O}}^{\prime },$$

where V_O_^×^ is the structural oxygen vacancy, $O_{V_{O}}^{\prime \prime }$ is the oxygen atom in the position of oxygen vacancy, and ${\left(\mathrm{OH}\right)}_{V_{O}}^{\prime }$ is the hydroxyl group on the structural oxygen vacancy site.

There are also a more complex crystal structures, such as intergrowth structures. For example, intergrowths with blocks of the structures containing coordinatively unsaturated polyhedra can be formed. Such structures have been known for a long time, but recent studies have shown that such structures are also capable of water incorporation and for exhibiting high proton conductivity. Such proton conductors with hexagonal perovskite related structures are still few in number.

Hexagonal perovskite Ba_7_Nb_4_MoO_20_ has been described as a pure ionic conductor with high proton conductivity 4.0 mS·cm^−1^ at 510 °C [37]. The structure of Ba_7_Nb_4_MoO_20_ can be represented as a cation-deficient 7H hexagonal perovskite derivative formed by an intergrowth of palmierite layers and 12R perovskite blocks [38]. Hydration of Ba_7_Nb_4_MoO_20_ occurs on the intrinsically oxygen-vacant palmierite-like layers with general composition Ba_3_M_2_O_8_ [39], and the formation of hydrated phases Ba_7_Nb_4_MoO_20_·0.79H_2_O has been proven by TG-measurement.

Murakami T. et al. have reported that the hexagonal perovskite with the composition of Ba_5_Er_2_Al_2_ZrO_13_ exhibited high proton conductivity 10^−3^ S·cm^−1^ at 300 °C [40]. An interesting feature of this phase is the sensitivity of conductivity to the presence of water vapor even at high temperatures: the ion conductivity at 800 °C in wet atmosphere was 132 times higher than that in the dry condition. According to neutron-diffraction data, the composition of the hydrated phase corresponded to Ba_5_Er_2_Al_2_ZrO_13.23_H_0.46_. The comparison of the conductivities in wet air (pH_2_O~0.02 atm) for the structural analogues Ba_5_R_2_Al_2_ZrO_13_ (R = Er, Yb, Tm, Dy, Lu) showed that all phases exhibited high conductivities; the highest values were realized for Er, Yb-containing samples, and the lower values up to 1.5 orders of magnitude at 300 °C were for the Lu-containing sample. The authors emphasized that the capability to proton transfer is due to the existence of intrinsically oxygen-deficient layers. The structural features of these phases were described by Shpanchenko R.V. et al. in 1995 [41]. The compound Ba_5_R_2_Al_2_ZrO_13_ (R = Er, Yb, Tm, Dy, Lu) adopts a hexagonal perovskite-related structure, consisting of triple-layer cubic perovskite blocks that are separated by an intrinsically oxygen-deficient hexagonal BaO□_2_–layer. The structure can be formally considered as intergrowth structures with alternating BaZrO_3_-type and β-Ba_2_ScAlO_5_-type structural blocks along the *c* axis (i.e., BaZrO_3_ + 2Ba_2_ScAlO_5_).

The structure of another compound Ba_5_In_2_Al_2_ZrO_13_ has been described by Shpanchenko R.V. et al. in 1994 [42]. It was shown that this structure can be considered as a result of the intergrowth of Ba_2_InAlO_5_-like blocks and BaZrO_3_-blocks along the *c*-axis. The main feature of the structure is joint occupation of two 4*f* positions by In and Al atoms and disordering of oxygen atoms and vacancies in BaO□_2_–layers (where the BaO_3_ and BaO□_2_ layers with disordered placement of the oxygen atoms and vacancies alternate along the *c*-axis; oxygen vacancies are localized in BaO□_2_ h-type layers). However, the electrical properties of this phase have not been studied and the possibility of proton transport has not been studied.

In this study, Ba_5_In_2_Al_2_ZrO_13_ phase and doped composition Ba_5_In_2.1_Al_2_Zr_0.9_O_12.95_ were prepared by the solid-state method, and their conductivity as a function of T and pO_2_ was investigated for the first time. The hydration processes, and the nature of oxygen-hydrogen groups and proton transport were examined for the first time.

## 2. Materials and Methods

The phase Ba_5_In_2_Al_2_ZrO_13_ and the doped sample Ba_5_In_2.1_Al_2_Zr_0.9_O_12.95_ were synthesized by the solid-state sintering method. The preliminary dried high-purity powders of BaCO_3_ (99.9999% purity, Vekton, RF), In_2_O_3_ (99.99% purity, Reachim, RF), Al_2_O_3_ (99.99% purity, Reachim, RF) and ZrO_2_ (99.99% purity, Reachim, RF) were used as starting materials. The stoichiometric amounts of these materials were mixed together in agate mortar and ground for 1 h. Powder mixtures were calcined at the temperature range of 800–1200 °C with heating steps of 100 °C for 24 h at each step with intermediate grindings.

X-ray powder diffraction was used to control phase purity on a ARL EQUINOX 3000 (Thermo Fisher Scientific, Waltham, MA, USA) diffractometer. Measurements were carried out at room temperature with Cu Kα radiation at the angle range 10–90° with a step of 0.024°. FullProf software was used for cell parameters calculations.

The morphology of the powder samples and the cationic composition of the ceramic samples were studied using a VEGA3 (Tescan, Brno, Czech Republic) scanning electron microscope (SEM) equipped with the AztecLive Standard Ultim Max 40 (Oxford Instruments, Oxford, UK) system for energy dispersive X-ray spectroscopy (EDS).

For investigations of electrical properties, the ceramic samples were prepared. The powders of Ba_5_In_2_Al_2_ZrO_13_ and Ba_5_In_2.1_Al_2_Zr_0.9_O_12.95_ were pressed into the pellets followed by sintering at 1450 °C for 24 h. Palladium-silver paste electrodes were painted on both sides of the sintered pellets and then fired at 900 °C for 3 h.

Electrochemical impedance spectroscopy measurements were carried out by the two-probe method with an Z-3000X (Elins, RF) frequency response analyzer (frequency range of 100 Hz–3 MHz). The impedance data analysis was carried out using the Zview software. The measurements were performed over the temperature range of 250–900 °C during cooling with a rate of 1 °C per min in dry and wet atmospheres. The data were collected on cooling in 20 °C interval steps with the equilibrium time of 30 min. Dry atmosphere (*p*H_2_O = 3.5·10^−5^ atm) was obtained by circulating gas through the phosphorous pentoxide powder P_2_O_5_. Wet atmosphere was provided by bubbling the gas through the saturated solution of potassium bromide KBr (*p*H_2_O = 1.92·10^−2^ atm). The humidity of gases was measured by a H_2_O-sensor (“Honeywell” HIH-3610, Freeport, TX, USA). Additionally, electric conductivity measurements were performed in a wide oxygen partial pressure range 10^−17^–0.21 atm. Oxygen partial pressure values were measured and controlled by an electrochemical sensor and pump from yttria-stabilized zirconia. The conductivity measurements were carried out under dry and wet atmospheres. Before taking a measurement, the impedance was monitored versus time to ensure that equilibrium was achieved.

Infrared (IR) spectroscopy measurements were used for identification of oxygen-hydrogen groups. Hydrated samples were analyzed by diffuse reflection technique on a Nicolet 6700 (Thermo Fisher Scientific, USA) FT-IR Spectrometer. All measurements were performed at room temperature. 

Hydrated samples were obtained for IR measurements according to the following scheme: the as-sintered powder sample was annealed at high temperature (1100 °C) in dry Ar in order to remove any protons or surface carbonates that the sample may have taken up during its exposure to ambient conditions. Then, the sample was cooled down to 150 °C under a flow of wet argon. 

Thermogravimetric (TG) measurements were carried out using a Pyris 1 (PerkinElmer, Waltham, MA, USA) TGA analyzer. The powder samples were heated at 1000 °C in dry argon atmosphere with a heating rate of 1 °C per minute, then the samples were cooled in wet argon (*p*H_2_O = 1.92·10^−2^ atm) with a rate of 1 °C per minute, and the data were collected upon cooling the samples to detect the mass gain during hydration and the temperature at which it occurs. The results were used for quantitative determination of proton concentration.

## 3. Results and Discussion

### 3.1. Structural Features, Phase Analysis and Composition Characterization

**Figure 1:**
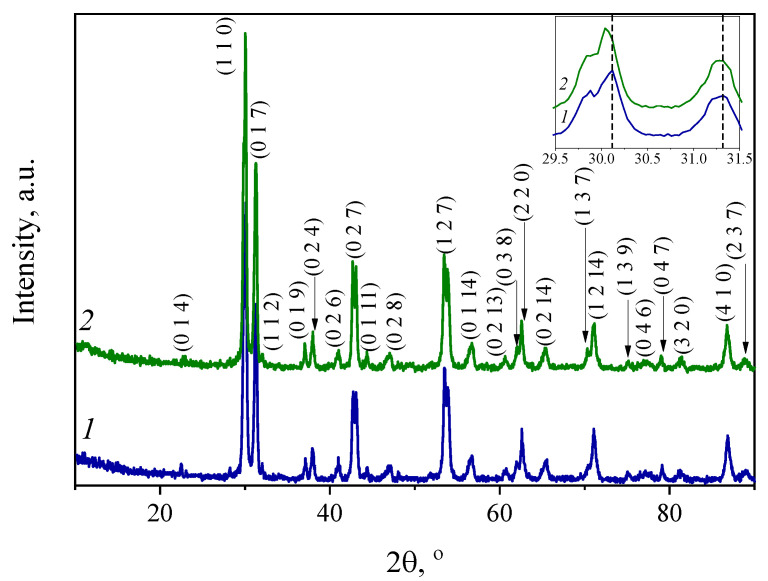
X-ray powder diffraction patterns for Ba_5_In_2_Al_2_ZrO_13_ (*1*) and Ba_5_In_2.1_Al_2_Zr_0.9_O_12.95_ (*2*); the insert shows an expanded view of the shifts of the diffraction peaks within 29.5–31.5°.

Figure 1 presents X-ray diffraction (XRD) patterns of Ba_5_In_2_Al_2_ZrO_13_ and Ba_5_In_2.1_Al_2_Zr_0.9_O_12.95_. As can be seen, obtained samples possess a primitive hexagonal structure. 

XRD patterns for both compounds are quite similar, thus it can be concluded that Ba_5_In_2_Al_2_ZrO_13_ and Ba_5_In_2.1_Al_2_Zr_0.9_O_12.95_ have identical structure and In_2_O_3_ dissolved in the matrix with formation of the solid-solution. The possible substitution reaction is the following:

(4) $$In_{2}O_{3}\overset{ZrO_{2}}{\to }2In_{Zr}^{\prime }+3O_{O}^{\times }+V_{O}^{••},$$

where In_Zr_^′^ represents In^3+^-cation at a tetravalent Zr-site.

**Figure 2:**
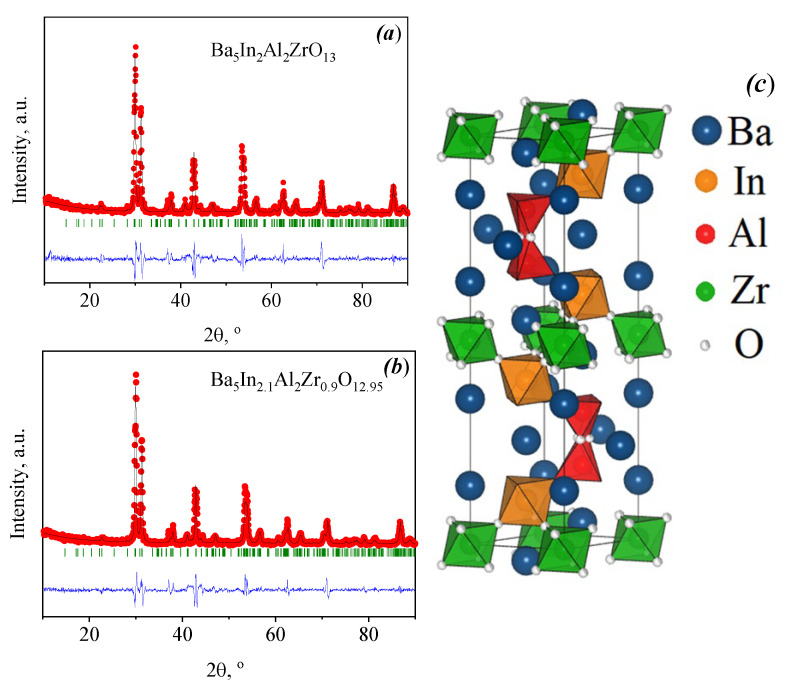
Rietveld profile fitting of powder X-ray diffraction pattern for Ba_5_In_2_Al_2_ZrO_13_ (**a**) and Ba_5_In_2.1_Al_2_Zr_0.9_O_12.95_ (**b**); observed (dots), calculated (line), difference (bottom) data, and angular positions of reflections (bars) are shown. The crystal structure of Ba_5_In_2_Al_2_ZrO_13_ (**c**).

Peak positions of Ba_5_In_2.1_Al_2_Zr_0.9_O_12.95_ had a small shift to the low-angle range in comparison with the undoped compound (Figure 1), thus the substitution of Zr^4+^ cation by In^3+^ led to an increase in the lattice parameters. Lattice parameters are *a* = 5.967(2) Å, *c* = 24.006(8) Å and *a* = 5.970(1) Å, *c* = 24.011(4) Å for Ba_5_In_2_Al_2_ZrO_13_ and Ba_5_In_2.1_Al_2_Zr_0.9_O_12.95_, respectively, space group *P6_3_/mmc*. This trend correlated with a higher ionic radius of indium ($r_{In^{3+}}$ = 0.80 Å [43]) in comparison with zirconium ($r_{Zr^{4+}}$ = 0.72 Å [43]). The examples of Rietveld profile fitting and crystal structure of Ba_5_In_2_Al_2_ZrO_13_ are shown in Figure 2.

**Figure 3:**
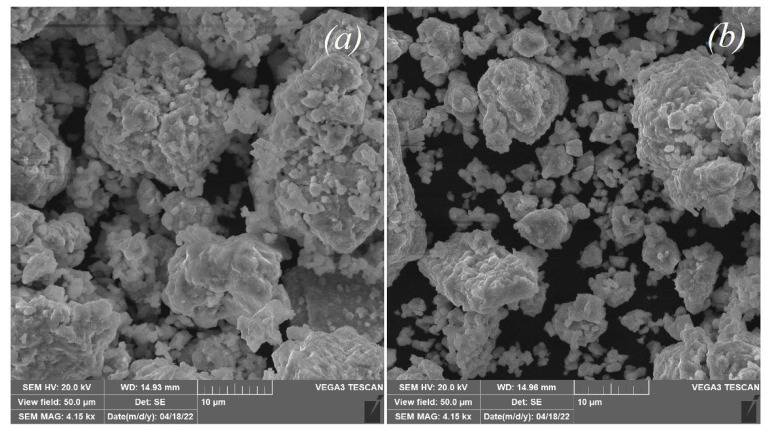
Scanning electron microscope (SEM) images for the powder samples Ba_5_In_2_Al_2_ZrO_13_ (**a**) and Ba_5_In_2.1_Al_2_Zr_0.9_O_12.95_ (**b**).

SEM images of the investigated powder compounds are presented in Figure 3. For both compounds, a grain size of about 10−15 μm was observed; these grains were agglomerated from the smaller crystallites. Substitution of zirconium by indium did not affect the grain sizes. In order to confirm these results regarding the average crystallite size, the additional estimation of the size from the powder diffraction patterns was made using the Sherrer’s formula:

(5) $$d=\frac{k\lambda }{\beta \cos\theta },$$

where *d* is crystallite size, k is the constant, λ is the wavelength, β indicates the full width at half maximum of the diffraction peak, and θ is the diffraction angle. The range for the crystallite size ranged from approximately 500 nanometers to 1 micron. This result is in good agreement with a typical SEM image (Figure 3), and confirms that the grains are agglomerates consisting of crystallites with an average size near 1 μm.

**Figure 4:**
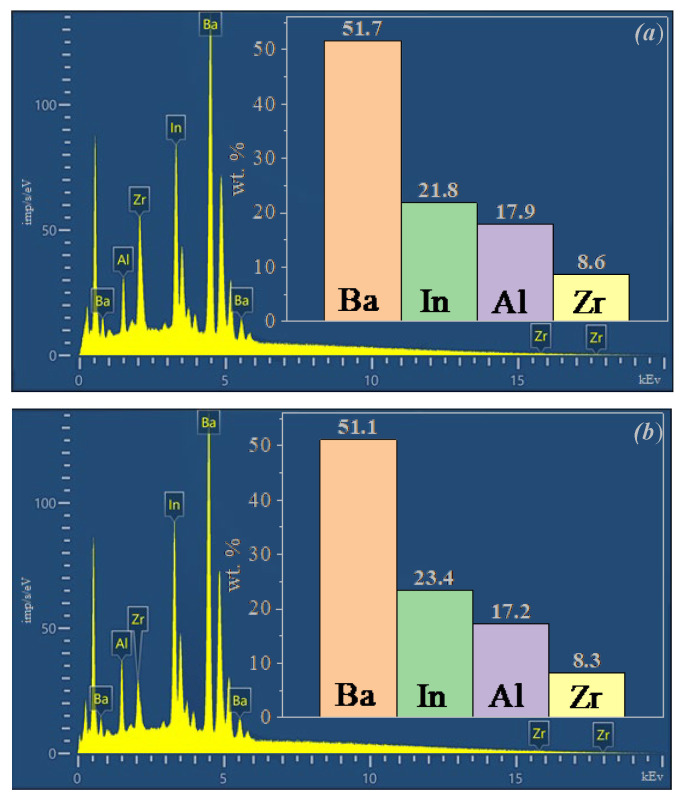
Energy dispersive X-ray spectroscopy (EDS) results and the element mass distribution histograms (at.%) for Ba_5_In_2_Al_2_ZrO_13_ (**a**) and Ba_5_In_2.1_Al_2_Zr_0.9_O_12.95_ (**b**).

The results of EDS analysis are shown in Figure 4. Experimentally obtained cation ratios are in good agreement with theoretical values. The comparison of theoretical and experimental compositions is shown in Table 1.

### 3.2. Hydration Behavior

**Figure 5:**
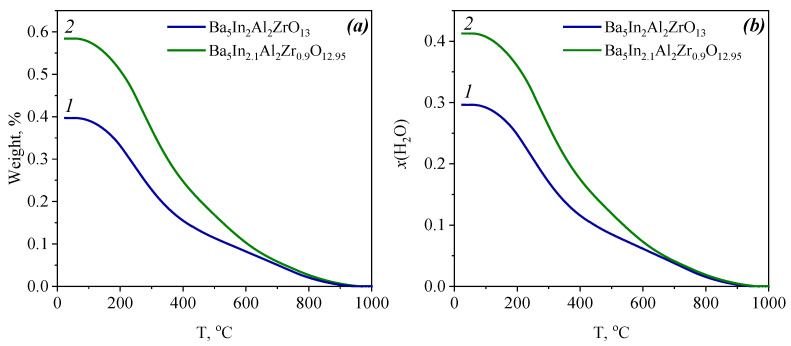
TG plots under cooling in wet Ar (*p*H_2_O = 1.92·10^−2^ atm) for Ba_5_In_2_Al_2_ZrO_13_·*x*H_2_O (*1*) and Ba_5_In_2.1_Al_2_Zr_0.9_O_12.95_ ·*x*H_2_O (*2*); % weight gain (**a**) and the hydration degree *x*H_2_O (**b**).

TG curves of Ba_5_In_2_Al_2_ZrO_13_ and Ba_5_In_2.1_Al_2_Zr_0.9_O_12.95_ during cooling in wet argon are shown in Figure 5a,b. Both samples exhibited a weight gain of about 0.4−0.6% as a result of hydration (Figure 5a). For convenience, the data are presented as mol H_2_O per formula unit, that is, as a temperature dependence of the degree of hydration *x*(H_2_O) (Figure 5b). In general, the shapes of the TG-curves for Ba_5_In_2_Al_2_ZrO_13_ and Ba_5_In_2.1_Al_2_Zr_0.9_O_12.95_ were quite similar. The mass changes were observed in a wide temperature range of 200−950 °C: the main mass changes occurred in the temperature range of 200−600 °C and insignificant mass change was observed in the temperature range of 600−950 °C. At the temperatures above 950 °C, the mass stabilization was observed. It could be said that mass changes were sufficiently monotonous, since there were not strongly pronounced jumps.

As shown above (Figure 2c), the structure of the investigated compound Ba_5_In_2_Al_2_ZrO_13_ can be described as intergrowth of two structural blocks of oxygen deficient phase Ba_2_InAlO_5_ and one structural block of oxygen completed perovskite BaZrO_3_ [42]; therefore, the compound Ba_5_In_2_Al_2_ZrO_13_ has a potential ability of water vapor incorporation due to presence of structural oxygen vacancies in their crystal lattice. Thus, the process of water incorporation into the structure can be described by the Equations (2) or (3). The doped phase has an additional number of oxygen vacancies due to acceptor doping. According to these structural features, the theoretical degree of hydration values should be 2 and 2.05 moles per formula unit for Ba_5_In_2_Al_2_ZrO_13_ and Ba_5_In_2.1_Al_2_Zr_0.9_O_12.95_, respectively. At the same time, it is known that the phase Ba_2_InAlO_5_ exhibits lower water intercalation capability: the hydration limit reaches 0.18 mol H_2_O per formula unit [44] due to corner sharing tetrahedra pairs [45]. In this aspect, the total degree of hydration of Ba_5_In_2_Al_2_ZrO_13_ should be ~0.4 mol. Experimentally obtained values of degree of hydration are *x*(H_2_O) = 0.30 and *x*(H_2_O) = 0.41 moles per formula unit for Ba_5_In_2_Al_2_ZrO_13_ and Ba_5_In_2.1_Al_2_Zr_0.9_O_12.95_, respectively. These values are close to the expected values. Hydration degree of In^3+^-doped phases is higher in comparison with the undoped compound. It can be explained by higher concentration of oxygen vacancies.

Comparing the obtained TG-data with the literature data obtained for the structural analog Ba_5_Er_2_Al_2_ZrO_13_, we can conclude that all these phases are characterized by close degrees of hydration. The H_2_O content in Ba_5_Er_2_Al_2_ZrO_13_ reached *x*~0.27 mol [40]. Another hexagonal perovskite Ba_7_Nb_4_MoO_20_ exhibited high hydration values of ~0.80 H_2_O molecules per formula unit [37,39]. The hydration is accompanied by transformation of tetrahedral NbO_4_ and MoO_4_ units in the palmierite-like layer to the NbO_6_ and MoO_6_ octahedra. In general, it can be concluded that the degrees of hydration will be determined by the coordination preferences of the cation, located in the unsaturated polyhedral, and the hydration energy.

### 3.3. The State of Oxygen-Hydrogen Groups

**Figure 6:**
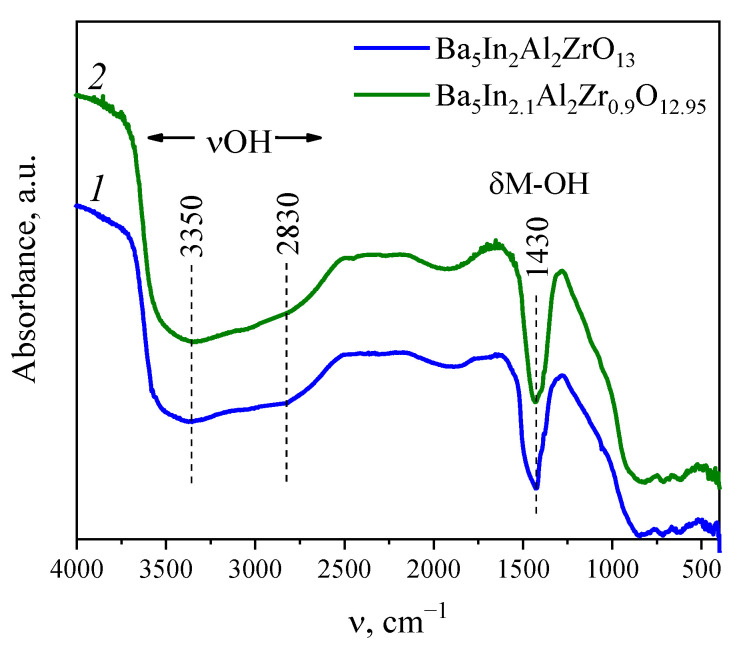
IR spectra of the hydrated samples Ba_5_In_2_Al_2_ZrO_13_ (*1*) and Ba_5_In_2.1_Al_2_Zr_0.9_O_12.95_ (*2*).

For IR investigations, the hydrated samples were performed as mentioned in the Experimental Section, and just before the IR measurements, the hydrated samples were annealed at ~150 °C to remove absorbed water. IR spectra are presented in Figure 6. Both spectra of Ba_5_In_2_Al_2_ZrO_13_ and Ba_5_In_2.1_Al_2_Zr_0.9_O_12.95_ have a similar shape. The presence of aa broad band in the range of 2500–3500 cm^−1^ confirms the existence of hydrogen-oxygen groups in the hydrated samples, since this frequency range is attributed to the oxygen-hydrogen stretching vibrations (υOH). The state of OH*_n_*-groups (i.e., H_3_O^+^, H_2_O, OH^−^) can be identified in the region of bending vibrations. The region below 1500 cm^−1^ corresponds to the bending vibrations of hydroxide groups, i.e., δM-OH. As such, the presence of the band with the frequency of 1400 cm^−1^ is attributed to the bending vibrations of the M-OH groups. The absence of bands at ~1600 and ~1700 cm^−1^ indicates the absence of water molecules and H_3_O^+^ ions, respectively. Thus, as for the majority of high-temperature protonic electrolytes with a perovskite-like structure, described in the literature, the main form of existence of oxygen-hydrogen groups in the investigated samples is OH^−^-groups.

Continuing the analysis of the range of stretching vibrations and taking into account the conclusion about the presence of OH^−^-groups, it can be said that the complex structure of the band in the range of 2500–3500 cm^−1^ indicates the presence of different OH^−^-groups. As it is seen, this band is a superposition of several components. The main maximum of this wide band is located near 3350 cm^−1^, which means that some OH^−^-groups should have a short length of the O–H bond (consequently the larger OH…O distance) and take part in weak hydrogen bonds. The lower frequency band located at ~2800 cm^−1^ indicates the presence of OH^−^-groups with a larger length of the bond and involved in stronger hydrogen bonds. There is also a band at the frequency of 1900 cm^−1^, corresponding to the mixed vibration; this band could not be used for the hydrogen-oxygen groups identification.

It can be concluded that substitution of zirconium by indium does not lead to any changing in the state of oxygen-hydrogen groups, and the OH^−^-group is the only form in which protons exist in the structure of the investigated phases. Accordingly, the composition of hydrated phases can be written as Ba_5_In_2_Al_2_ZrO_13_·0.30H_2_O≡Ba_5_In_2_Al_2_ZrO_12.7_(OH)_0.6_ and Ba_5_In_2.1_Al_2_Zr_0.9_O_12.95_·0.41H_2_O≡Ba_5_In_2.1_Al_2_Zr_0.9_O_12.54_(OH)_0.82_. At least two types of OH^−^-groups are present in the structure which participate in different hydrogen bonds. The presence of different OH^−^-groups suggests their different thermal stability, which is manifested on the TG-curves as the effects of mass changes in different temperature ranges.

### 3.4. Electrochemical Properties

**Figure 7:**
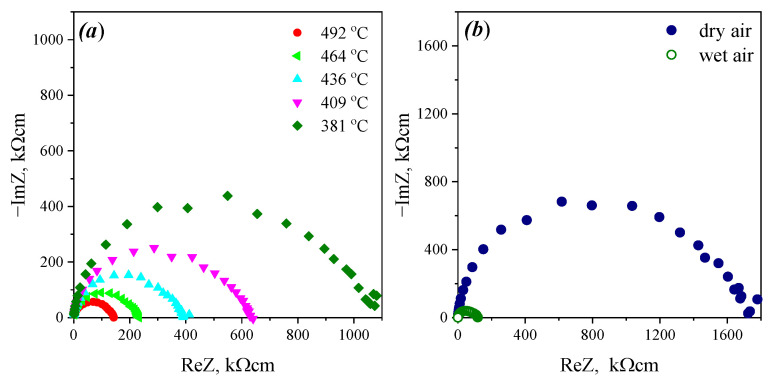
Impedance spectra for Ba_5_In_2_Al_2_ZrO_13_ at different temperatures in dry air (**a**), and the comparison in dry (*p*H_2_O = 3.5·10^−5^ atm) and wet air (*p*H_2_O = 1.92·10^−2^ atm) at 350 °C (**b**).

Figure 7 shows the typical complex impedance plots of the Ba_5_In_2_Al_2_ZrO_13_ sample in dry atmosphere at different temperatures (*a*), and in comparison, at 350 °C in dry and wet atmospheres (*b*). As can be seen, the general shape of impedance spectra in dry and wet atmospheres and at different temperatures was quite similar. The Nyquist plots (ImZ imaginary part vs. ReZ real part of the impedance) indicated the presence of at least two relaxation processes in the region of the main studied frequencies; as it is seen, the spectra can be characterized by two overlapping semicircles. Besides, a small contribution from the third semicircle in the low frequency region was also present. It is known that in polycrystalline ion-conducting materials, the relaxation processes can be represented by at least three contributions: the bulk of the material (*f*_b_), the grain boundaries (*f*_gb_), or the electrode processes (*f*_el_), where *f*_b_ > *f*_gb_ > *f*_el_ [46]. Any other relaxation processes in the impedance spectra were not found in a wide range of studied frequencies of 100 Hz–3 MHz.

A primary analysis of the capacities for these observed contributions can provide information about the nature of these processes [47]. The calculated values of capacitance attributed to the observed third arc were about 10^−6^ F, which is typical for the processes at the interface between electrodes and ceramic samples. Part of this third semicircle was visible in a limited temperature range (this relaxation process is not discussed further, because the electrode impedance was not the aim of this study). Capacitance values attributed to the first and second arcs were about 10^−11^ and 10^−10^ F, respectively; thus, the first arc should be corresponded to the bulk contribution and the second arc to the grain boundary contribution. It should be noted that the second semicircle is much smaller than the first one, hence it could be expected that grain boundaries resistance effect on material’s total resistance should be insignificant. 

**Figure 8:**
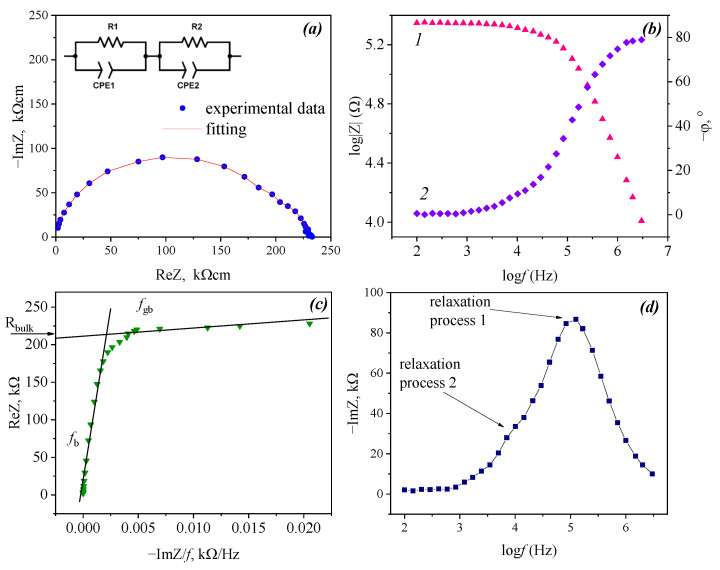
The example of the impedance data fitting for Ba_5_In_2_Al_2_ZrO_13_ and corresponding equivalent circuit, R1, R2, CPE1 and CPE2 represent the bulk resistance, grain-boundary resistance, constant phase element of the bulk and constant phase element of the grain-boundaries (wet air *p*H_2_O = 1.92·10^−2^ atm, 464 °C) (**a**); the impedance data shown in the coordinates of the logarithm of the impedance magnitude (1) and phase-shift (2) vs. the logarithm of the frequency (**b**). The impedance spectrum shown in the real part of impedance ReZ vs. ImZ/*f* representation (**c**). Impedance data shown as imaginary part ImZ vs. the logarithm of the frequency (**d**).

At the same time, it is known that alternative formalisms of data presentation can yield additional information that is not easily accessible from the impedance complex plane [47,48]. For example, impedance data can be represented as Bode plots in various coordinates. To identify and separate the different relaxation processes, we presented the data also in coordinates of the logarithm of the impedance magnitude and phase-shift vs. the logarithm of the frequency (Figure 8b). In the Bode diagram, the difference of the two kinds of relaxation processes cannot be discerned so clearly. As such, the data can then be plotted in different ways. The appearance of two impedance contributions can be better seen from representation as the real part of impedance (ReZ) vs. imaginary part divided by the frequency (ImZ/*f*), as shown in Figure 8c. This plot shows two linear regions, indicating the presence of two relaxation processes. The linear region in such plots represents a relaxation process, with the *y*-intercept of the line giving the relaxation frequency and a characteristic resistance, respectively [48]. In Figure 8d, the impedance spectroscopic data of Ba_5_In_2_Al_2_ZrO_13_ recorded at 464 °C in the imaginary part ImZ vs. the logarithm of the frequency representation were shown. The contributions of two relaxation processes can be distinguished.

Thus, we can conclude that the impedance data obtained can be adequately represented as two relaxation processes and modeled as two parallel RCPE elements. An example of the fitting of the experimental data and equivalent circuit is shown in Figure 8a. For further discussions, the calculated values of the bulk resistance were used.

**Figure 9:**
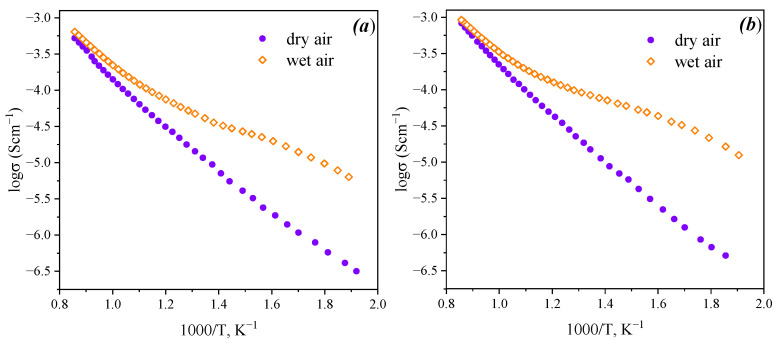
Temperature dependence of total conductivity in dry (*p*H_2_O = 3.5·10^−5^ atm) and wet (*p*H_2_O = 1.92·10^−2^ atm) air for Ba_5_In_2_Al_2_ZrO_13_ (**a**) and Ba_5_In_2.1_Al_2_Zr_0.9_O_12.95_ (**b**).

Plots of the temperature dependencies of bulk conductivities for Ba_5_In_2_Al_2_ZrO_13_ and Ba_5_In_2.1_Al_2_Zr_0.9_O_12.95_ are presented in Figure 9. It should be noted that across the whole temperature range the conductivity values in wet atmosphere were higher in comparison with dry atmosphere. The difference in conductivity values in wet and dry atmospheres increases with decreasing temperature, and at temperatures below 350 °C, the difference becomes more than one order. The difference between conductivity values in dry and wet atmospheres becomes insignificant at temperatures above 850 °C, although the values in wet atmosphere are still higher than in dry. These results confirm the possibility of the appearance of proton transport in the studied phases.

The values of conductivity of Ba_5_In_2.1_Al_2_Zr_0.9_O_12.95_ are higher in comparison with undoped compound in both dry and wet atmospheres. Differences in total conductivity values in wet and dry atmosphere are also higher in the case of In-doped compound. This can be explained by the oxygen vacancies formation due to acceptor doping, that can be described by the quasi-chemical Equation (4). As can be seen from this equation, the substitution of zirconium by indium leads to the formation of the charged oxygen vacancies V_O_^••^. In wet air, the proton charge carrier formation occurs due to water incorporation, so conductivities increase.

In order to determine the type of dominant carrier and partial conductivities, the measurements of conductivity as a function of oxygen partial pressure were made.

**Figure 10:**
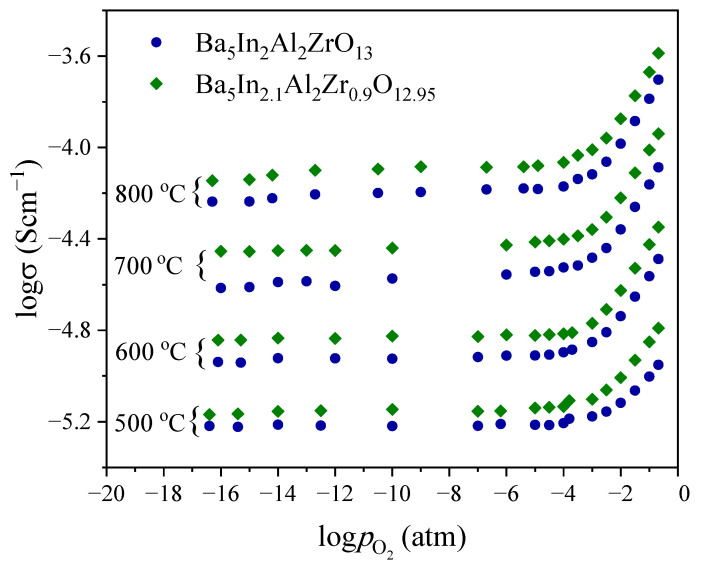
Oxygen partial pressure dependence of total conductivity in dry atmosphere (*p*H_2_O = 3.5·10^−5^ atm) for Ba_5_In_2_Al_2_ZrO_13_ and Ba_5_In_2.1_Al_2_Zr_0.9_O_12.95_.

The dependencies of conductivity as a function of oxygen partial pressure in dry atmosphere are presented in Figure 10. It can be seen that the values of conductivity decrease with reducing oxygen partial pressure from 0.21 to 10^−4.5^ atm. This behavior indicates the presence of some contribution of p-type conductivity σ*_h_*; the oxygen incorporation process with formation of holes can be expressed by the equation:

(6) $$V_{O}^{\times }+\frac{1}{2}O_{2}↔2h^{•}+O_{V_{O}}^{\prime \prime },$$

where *h^•^* is hole, and $O_{V_{O}}^{\prime \prime }$ is the oxygen atom in the position of oxygen vacancy.

At the oxygen partial pressure range of 10^−17^–10^−4.5^ atm, the conductivity dependence exhibits plateau. In this oxygen partial pressure range, the ionic defects dominate while concentrations of electron defects are negligible (σ^dry^_ion_ = σ_O_^2−^). This ionic conductivity plateau is attributed to the intrinsic oxygen vacancies present in the structure. With a change in the temperature, the general view of the dependences does not change, but a slight decrease in the positive slope of the dependences with decreasing temperature can be noted.

The general view of the dependences for both samples is similar; somewhat higher conductivity values are observed for the doped sample Ba_5_In_2.1_Al_2_Zr_0.9_O_12.95_ in comparison with Ba_5_In_2_Al_2_ZrO_13_ over the whole investigated oxygen partial pressure range. The increase in ionic conductivity (plateau region) is due to an increase in the concentration of oxygen vacancies during doping. Moreover, it should be noted that, in the range from 10^−4.5^ to 0.21 atm, the conductivity of doped samples is also higher. This means that the hole conductivity is higher in the Ba_5_In_2.1_Al_2_Zr_0.9_O_12.95_ in comparison with the undoped compound. As it was mentioned earlier, the doping process leads to formation of the charged oxygen vacancies V_O_^••^; hence, in the range of high oxygen partial pressures the process of oxygen incorporation may also produce a hole, as can be showed by the equation:

(7) $$V_{O}^{••}+\frac{1}{2}O_{2}↔2h^{•}+O_{O}^{\times },$$

where V_O_^••^ is oxygen vacancy, O_O_^×^ is the oxygen at the regular oxygen site, and *h^•^* is the hole.

Thus, at a high oxygen partial pressure range, the increase in the concentration of oxygen vacancies may also increase the concentration of holes, that will raise the values of hole conductivity and, as a result, it’s contribution to the total conductivity value. 

Thus, in air (*p*O_2_ = 0.21 atm), the total conductivity of both investigated phases is characterized by a mixed type, and in general, the case can be represented as:

(8) σ=σion+σh=σion+σ0pO214,

where σ_ion_ is ion conductivity, σ*_h_* is hole conductivity, *p*O_2_ is oxygen partial pressure, and σ^0^ is hole conductivity at *p*O_2_ = 1 atm.

**Figure 11:**
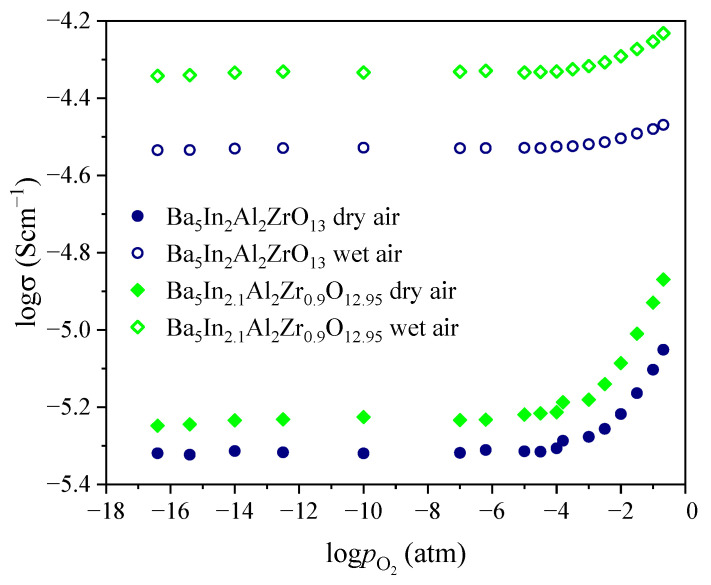
Oxygen partial pressure dependence of total conductivity at 500 °C in dry (*p*H_2_O = 3.5·10^−5^ atm) and wet (*p*H_2_O = 1.92·10^−2^ atm) atmospheres for Ba_5_In_2_Al_2_ZrO_13_ and Ba_5_In_2.1_Al_2_Zr_0.9_O_12.95_.

**Figure 12:**
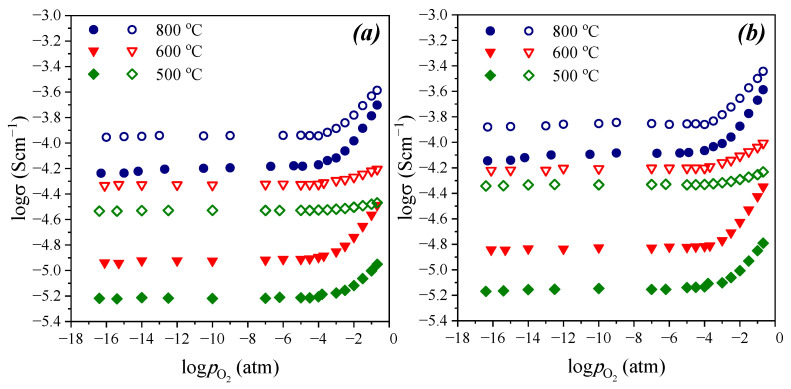
Oxygen partial pressure dependencies of total conductivity in dry (*p*H_2_O = 3.5·10^−5^ atm) and wet (*p*H_2_O = 1.92·10^−2^ atm) atmospheres for Ba_5_In_2_Al_2_ZrO_13_ (**a**) and Ba_5_In_2.1_Al_2_Zr_0.9_O_12.95_ (**b**).

Figure 11 and Figure 12 show oxygen partial pressure dependences of total conductivity in dry and wet atmosphere in comparison. As can be seen, across the whole oxygen partial pressure range, the values of conductivity in wet atmosphere are higher in comparison with dry atmosphere. Conductivity increase in wet atmosphere is due to the contribution of proton conductivity (σ^wet^_ion_ = σ_O_^2−^ + σ_H_). It should be noted that differences in conductivity values in dry and wet atmospheres increases with decreasing temperature. This is due to hydration process which leads to proton concentration increase. Besides, the difference between dry and wet atmospheres increases with oxygen partial pressure decrease, and at 500 °C, the differences are about one order of magnitude at the plateau region. In general, it can be said that below 500 °C, the samples were characterized by the dominant ionic type of conductivity over the whole investigated *p*O_2_ range. Accordingly, in air (*p*O_2_ = 0.21 atm), they exhibited the dominant proton conductivity.

**Figure 13:**
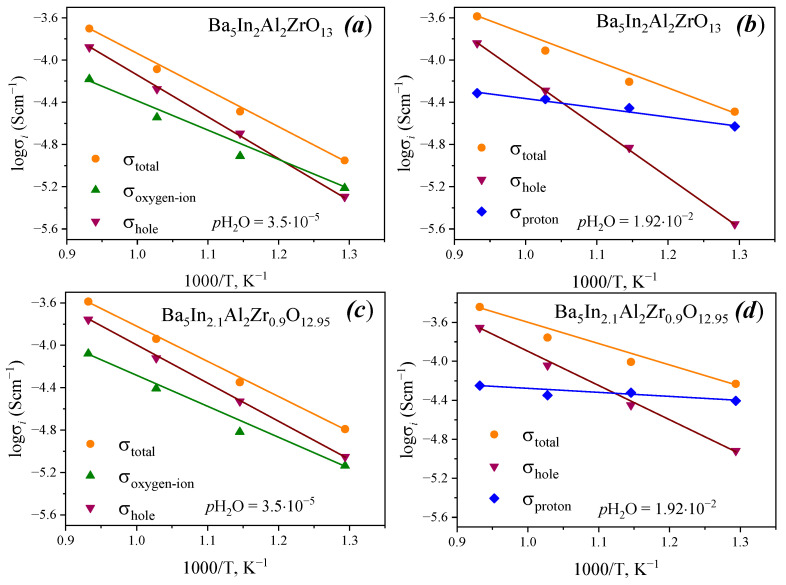
Partial conductivities for Ba_5_In_2_Al_2_ZrO_13_ in dry (**a**) and wet (**b**) atmospheres, and for Ba_5_In_2.1_Al_2_Zr_0.9_O_12.95_ in dry (**c**) and wet (**d**) atmospheres.

According to the data on conductivity dependences vs. oxygen partial pressure, the partial conductivities were calculated in air. For dry air, the values of oxygen-ion conductivities were defined as the conductivity values in the plateau region; the values of hole conductivity were defined by subtracting the ionic conductivity from the total conductivity at *p*O_2_ = 0.21 atm (in accordance with Equation (8)), i.e., σ*_h_* = σ(0.21 atm) − σ_ion_. Proton conductivity values were calculated by subtraction of ionic conductivity in wet atmospheres (the plateau for wet conditions) from the values of oxygen-ion conductivity in dry air (the plateau for dry conditions), i.e., σ_H_ = σ^wet^_ion_ − σ^dry^_O_^2−^ (assuming that the oxygen ion conductivity is independent of the proton concentration). The values of hole conductivity were defined as a difference of total conductivity value at oxygen pressure 0.21 atm and of the value of ion conductivity. Temperature dependencies of partial conductivities are shown in Figure 13.

As can be seen from Figure 13, in dry air, over the whole temperature range, the values of hole conductivity are higher than oxygen-ion conductivity. Only at 500 °C for Ba_5_In_2_Al_2_ZrO_13_ did oxygen-ion conductivity values become higher than values of hole conductivity; for Ba_5_In_2.1_Al_2_Zr_0.9_O_12.95_ at this temperature, the hole conductivity value was still higher. In wet air below 600 °C, both compounds exhibit dominant proton conductivity.

Table 2 presents the values of activation energies E_a_ for oxygen-ion and proton conductivities, and the pre-exponential terms A, which were calculated according to the Arrhenius equation:

(9) $$\sigma T=A\;\exp(\frac{-E_{a}}{kT}),$$

where A is pre-exponential term, *k* is the Boltzmann constant, and T is absolute temperature. As it is seen, activation energies of oxygen-ion conductivities were the same for both samples, and these values are typical for oxygen-ion transport for hexagonal structures. For example, the activation energy for the compound Ba_5_Er_2_Al_2_ZrO_13_ with close structure was 0.58 eV [40]. The value of the pre-exponential term for the doped sample Ba_5_In_2.1_Al_2_Zr_0.9_O_12.95_ was higher than for Ba_5_In_2_Al_2_ZrO_13_, and this may be due to the increase in the unit cell size of the doped sample, and hence the increase in the interatomic distance and, consequently, the distance the ion mean free path. This is a favorable factor for ion transport.

The value of activation energies E_a_ of proton conductivity for both samples are quite low; the activation energy for Ba_5_In_2.1_Al_2_Zr_0.9_O_12.95_ is lower than for Ba_5_In_2_Al_2_ZrO_13_, thus the process of protons migration is facilitated in In-doped compound. The decrease in the pre-exponential factor was compensated by the decrease in activation energy, and thus the proton conductivity increased during doping. It is known that proton transport in oxides, occurring according to the Grotthuss mechanism, is characterized by activation energies of ~0.5 eV. These values are typical for many perovskites and related structures, the comparison of activation energies E_a_ of proton conductivity for various structures is presented in [49]. It should be emphasized that proton transport in hexagonal perovskites with general formula Ba_5_M^3+^_2_Al_2_ZrO_13_ is realized with lower activation energies. The activation energies for conductivity in wet air below 450 °C were 0.29, 0.33, 0.36 and 0.40 eV for M = Tm, Dy, Yb and Er [40]. These values are comparable with the values obtained in this work for In-containing samples.

**Figure 14:**
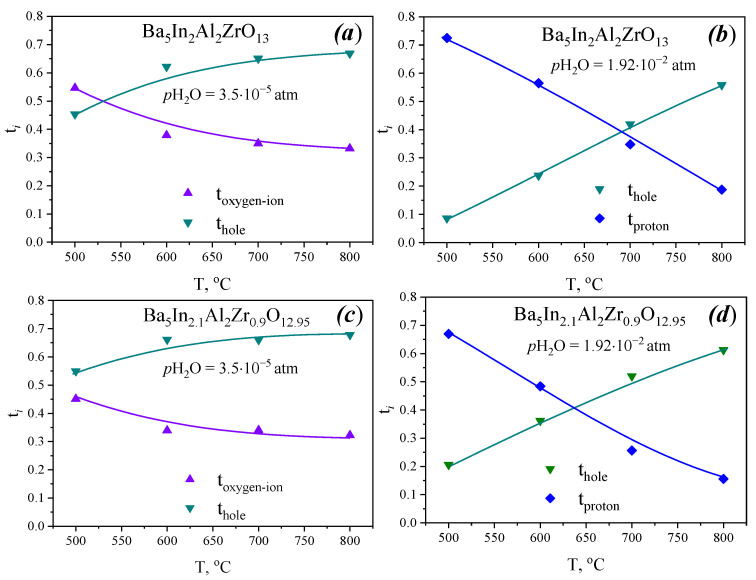
Transport numbers for Ba_5_In_2_Al_2_ZrO_13_ in dry (**a**) and wet (**b**) air, and for Ba_5_In_2.1_Al_2_Zr_0.9_O_12.95_ in dry (**c**) and wet (**d**) air.

In addition, the values of transport numbers were calculated. For each type of partial conductivity, transport numbers t*_i_* were calculated according to the following equation:

(10) $$t_{i}=\frac{\sigma_{i}}{\sigma },$$

where σ*_i_* is partial conductivity, and σ is total conductivity in dry or wet atmospheres. Temperature dependencies of transport numbers are shown in Figure 14.

As can be seen, in dry atmosphere at 500 °C, both compounds are mixed hole-oxygen-ion conductors. With decreasing temperature, the contribution of oxygen-ionic conductivity increases insignificantly. At all temperatures, the contribution of hole conductivity for Ba_5_In_2.1_Al_2_Zr_0.9_O_12.95_ is higher in comparison with Ba_5_In_2_Al_2_ZrO_13_.

In wet atmosphere with decreasing temperature, proton transport numbers rise due to the hydration process, and at 600 °C, the values of total conductivity are mainly determined by proton transport.

**Figure 15:**
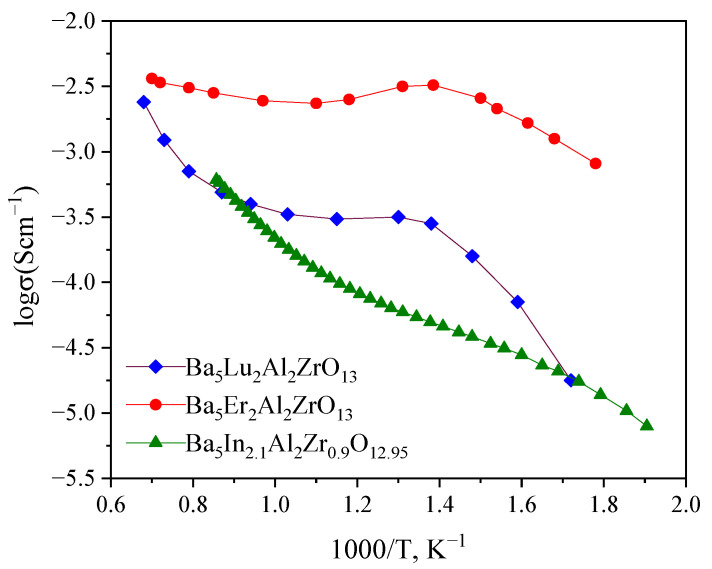
Temperature dependencies of total conductivity in wet air (*p*H_2_O = 1.92·10^−2^ atm) for Ba_5_Er_2_Al_2_ZrO_13_ [40], Ba_5_Lu_2_Al_2_ZrO_13_ [40] and Ba_5_In_2.1_Al_2_Zr_0.9_O_12.95_.

The comparison of the temperature dependencies of total conductivity in wet air for Ba_5_Er_2_Al_2_ZrO_13_ [40], Ba_5_Lu_2_Al_2_ZrO_13_ [40] and investigated phase Ba_5_In_2.1_Al_2_Zr_0._9O_12.95_ is shown in Figure 15. As can be seen, the Er-containing sample exhibited the highest conductivities. The investigated phase was characterized by a conductivity close to that of the Lu-containing phase. We believe that this is due to the closer radii of In^3+^ (*r* = 0.80 Å) [43] and Lu^3+^ (*r* = 0.86 Å) [43], compared to Er^3+^ (*r* = 0.89 Å) [43]. Probably, for the Er^3+^-containing sample, the optimal migration volume for oxygen-ion and proton transport is realized.

Thus, we can conclude that the studied phases Ba_5_In_2_Al_2_ZrO_13_ and Ba_5_In_2.1_Al_2_Zr_0.9_O_12.95_ are promising proton conductors, and acceptor doping is a promising way to optimize proton conductivity in hexagonal perovskites.

## 4. Conclusions

The hexagonal perovskite Ba_5_In_2_Al_2_ZrO_13_ and In^3+^-doped phase Ba_5_In_2.1_Al_2_Zr_0.9_O_12.95_ were prepared by the solid-state synthesis method. Both phases had a hexagonal structure with a space group of *P6_3_/mmc* and the lattice parameters *a* = 5.967(2) Å, *c* = 24.006(8) Å and *a* = 5.970(1) Å, *c* = 24.011(4) Å, respectively. The substitution of Zr^4+^ by a larger in size In^3+^ led to an increase in the lattice parameters.

TG-measurements confirmed that the phases were capable of incorporating water and forming hydrated phases of the compositions Ba_5_In_2_Al_2_ZrO_12.7_(OH)_0.6_ and Ba_5_In_2.1_Al_2_Zr_0.9_O_12.54_(OH)_0.82_. The concentration of protons in the doped phase was higher due to the appearance of an additional amount of oxygen vacancies.

IR-studies confirmed the presence of different OH^−^-groups, which participate in different hydrogen bonds, and, as a result, have different thermal stability.

The electrical conductivities of both samples under wet (*p*H_2_O = 1.92·10^−2^ atm) and dry (*p*H_2_O = 3.5·10^−5^ atm) atmospheres with different oxygen partial pressure *p*O_2_ values were investigated using the *ac* impedance spectroscopy technique. The activation energy values for oxygen-ions and protons were found to be 0.64 eV and 0.27 eV for undoped sample Ba_5_In_2_Al_2_ZrO_13_, and 0.65 eV and 0.19 eV for the In^3+^-doped sample. Both phases in dry air showed mixed (oxygen-ion and hole) type of conductivity. Ionic transport numbers reached 0.3–0.5 in the investigated temperature range of 500–800 °C. The acceptor doping made it possible to increase the oxygen-ionic conductivity but not significantly; the differences reached 0.1 order of magnitude. Both phases exhibited proton conductivity, which dominated in wet air below 500 °C. The difference between dry and wet atmospheres increased with decreasing oxygen partial pressure, and at 500 °C, the differences were about one order of magnitude at a plateau region. The value of activation energies E_a_ of proton conductivity for both samples were quite low, the activation energy for Ba_5_In_2.1_Al_2_Zr_0.9_O_12.95_ (0.19 eV) was lower than for Ba_5_In_2_Al_2_ZrO_13_ (0.27 eV). The increase in the proton conductivity for the doped phase is explained by the higher concentrations of protons and the increase in their mobility.

## Figures and Tables

**Table 1:** Elemental composition determined by the EDS for the samples Ba_5_In_2_Al_2_ZrO_13_ and Ba_5_In_2.1_Al_2_Zr_0.9_O_12.95_.

Compound	Theoretical Values, at.%				EDS Analysis, at.%			
	Ba	In	Al	Zr	Ba	In	Al	Zr
Ba_5_In_2_Al_2_ZrO_13_	50	20	20	10	51.7	21.8	17.9	8.6
Ba_5_In_2.1_Al_2_Zr_0.9_O_12.95_	50	21	20	9	51.1	23.4	17.2	8.3

**Table 2:** Comparison of the activation energy and pre-exponential term of Ba_5_In_2_Al_2_ZrO_13_ and Ba_5_In_2.1_Al_2_Zr_0.9_O_12.95_ for oxygen-ion and proton conductivities.

Compound	A(O^2−^), Ω^−1^cm^−1^K	E_a_(O^2−^), eV	A(H^+^), Ω^−1^cm^−1^K	E_a_(H^+^), eV
Ba_5_In_2_Al_2_ZrO_1__3_	63 ± 1	0.64 ± 0.01	0.54 ± 0.01	0.27 ± 0.01
Ba_5_In_2.__1_Al_2_Zr_0.__9_O_12.__95_	106 ± 1	0.65 ± 0.01	0.36 ± 0.01	0.19 ± 0.01

## Data Availability

The reported data is available from the corresponding authors on a reasonable request.
